# Treatment of Myopia with Atropine 0.125% Once Every Night Compared with Atropine 0.125% Every Other Night: A Pilot Study

**DOI:** 10.3390/jcm12165220

**Published:** 2023-08-10

**Authors:** Zi-Rong Chen, Shin-Chieh Chen, Tsung-Yao Wan, Lan-Hsin Chuang, Hung-Chi Chen, Lung-Kun Yeh, Yu-Kai Kuo, Pei-Chang Wu, Yun-Wen Chen, Ing-Chou Lai, Yih-Shiou Hwang, Chun-Fu Liu

**Affiliations:** 1 College of Medicine, Chang Gung University, Taoyuan 333, Taiwan; mpq791@cgmh.org.tw (Z.-R.C.); u111218201@cmu.edu.tw (T.-Y.W.); c571019@cgmh.org.tw (L.-H.C.); mr3756@cgmh.org.tw (H.-C.C.); yeh4156@cgmh.org.tw (L.-K.Y.); ken097551503@cgmh.org.tw (Y.-K.K.); wpc@adm.cgmh.org.tw (P.-C.W.); curiouscat@cgmh.org.tw (Y.-W.C.); lai1@cgmh.org.tw (I.-C.L.); ejubibi@cgmh.org.tw (Y.-S.H.); 2 Department of Environmental and Occupational Medicine, National Taiwan University Hospital, Taipei 100, Taiwan; 122232@ntuh.gov.tw; 3 Institute of Environmental and Occupational Health Sciences, College of Public Health, National Taiwan University, Taipei 100, Taiwan; 4 Department of Ophthalmology, Chang Gung Memorial Hospital, Keelung 204, Taiwan; 5 Department of Ophthalmology, Chang Gung Memorial Hospital, Linkou, Taoyuan 333, Taiwan; 6 Center for Tissue Engineering, Chang Memorial Hospital, Linkou, Taoyuan 333, Taiwan; 7 Department of Ophthalmology, Kaohsiung Chang Gung Memorial Hospital, Kaohsiung 833, Taiwan; 8 Department of Ophthalmology, Chang Gung Memorial Hospital, Chiayi City 613, Taiwan; 9 Department of Ophthalmology, Chang Gung Memorial Hospital, Xiamen Branch, Xiamen 361000, China; 10 Program in Molecular Medicine, National Yang Ming University, Taipei 112, Taiwan

**Keywords:** myopia, atropine, atropine frequency, myopia control, atropine adverse effect

## Abstract

(1) Purpose: To investigate the efficacy of myopia treatment in children using atropine 0.125% once every two nights (QON) compared with atropine 0.125% once every night (HS). (2) Methods: This retrospective cohort study reviewed the medical records of two groups of children with myopia. Group 1 comprised children treated with atropine 0.125% QON, while group 2 included children treated with atropine 0.125% HS. The first 6 months of data of outcome measurements were subtracted as washout periods in those children undergoing both atropine QON and HS treatment. The independent t-test and Pearson’s chi-square test were used to compare the baseline clinical characteristics between the two groups. A generalized estimating equations (GEE) model was used to determine the factors that influence treatment effects. (3) Results: The average baseline ages of group 1 (38 eyes from 19 patients) and group 2 (130 eyes from 65 patients) were 10.6 and 10.2 years, respectively. There were no significant differences in axial length (AL) or cycloplegic spherical equivalent (SEq) at baseline or changes of them after 16.9 months of follow-up. GEE showed that the frequency of atropine 0.125% use has no association with annual AL (QON vs. HS: 0.16 ± 0.10 vs. 0.18 ± 0.12) and SEq (QON vs. HS: −0.29 ± 0.44 vs. −0.34 ± 0.36) changes in all children with myopia. It also showed that older baseline age (B = −0.020, *p* < 0.001) was associated with lesser AL elongation. (4) Conclusion: The treatment effects of atropine 0.125% HS and QON were similar in this pilot study. The use of atropine 0.125% QON may be an alternative strategy for children who cannot tolerate the side effects of atropine 0.125% HS. This observation should be confirmed with further large-scale studies.

## 1. Introduction

Epidemiological studies have demonstrated a global increase in myopia, particularly in certain regions such as parts of China and other Southeast Asian countries. In these areas, myopia has become the most common refractive error among children, affecting between 69% and 86% of 15-year-olds [1]. However, the prevalence of myopia in other countries, such as Colombia in South America, is not as high, with a reported rate of 14.7% among 15-year-old adolescents [2]. Similarly, among native Africans, the pooled rate of myopia among 15-year-olds is low (5.5%) [1,2,3,4,5]. This progressive ocular disease primarily results from excessive axial length (AL) elongation during school age [3,5,6]. Excessive axial elongation may cause damage to the neuroretina of the eye, such as myopic maculopathy, high myopia-associated optic neuropathy, retinal detachment, cataracts, and glaucoma, which can lead to irreversible vision loss [3,5]. Therefore, management of myopia is necessary to prevent complications. AL and cycloplegic refraction changes are the two most important parameters for monitoring myopia progression and treatment effect [3,5,7]. Of these two parameters, changes in AL are more convenient and reliable due to less influence by accommodation (complete cycloplegia in children is not easy to achieve). Additionally, they have a more direct connection to pathological myopia [5,8]. It has been suggested that AL can be inferred from keratometry and refractive data. However, this is imprecise and must therefore be measured with a biometer directly [9].

There are various interventions for controlling myopia progression in children [10]. Of them, topical atropine is the treatment with the most evidence of slowing axial myopia progression [8,10]. Unfortunately, some patients cannot tolerate the side effects of atropine, a muscarinic antagonist, such as photophobia, blurring at near-sight range, dizziness, nausea, and loss of balance [10]. These side effects may decrease children’s compliance with atropine use. Fortunately, lower doses of atropine (i.e., 0.1%, 0.05%, and 0.01%) were found to not only have fewer side effects than higher doses (i.e., 1% and 0.5%) but also have adequate efficacy in myopia control [11,12]. Lower-dose atropine may also have lower rebound effects when patients stop using atropine [11].

In Taiwan, the most commonly prescribed concentration for myopia control is 0.125% atropine because it is the lowest dose covered by the national health insurance [13]. However, some patients may experience side effects with this concentration. Reducing the frequency of atropine use from once every night (HS) to once every two nights (QON) can be considered a reasonable alternative. Some studies have explored the use of high-concentration atropine, i.e., 1%, administered once, twice, or thrice per week, or even once a month, and have shown good efficacy [14,15,16,17]. However, to date, no studies are addressing the efficacy of myopia control when the frequency of lower-dose atropine treatment is reduced to less than once a day. Therefore, we conducted this study to explore the efficacy of myopia control using atropine 0.125% QON compared to atropine 0.125% HS [9,14,15,16,17].

## 2. Materials and Methods

### 2.1. Subjects

This retrospective cohort study enrolled subjects who were myopic children aged from 5 to 18 years, with cycloplegic spherical equivalent (SEq) values in the range of −0.25 D to −6 D, undergoing atropine 0.125% treatment. Data were obtained from the Atropine Continuous Follow-Up (ATCFU) open database, which contains detailed examination data of patients under atropine treatment for myopia control in Keelung Chang Gung Memorial Hospital, Keelung, Taiwan, since January 2017. The study protocol was approved by the Institutional Review Board of Keelung Chang Gung Memorial Hospital (Approval No.: 202001771B0) and followed the tenets of the Declaration of Helsinki.

### 2.2. Inclusion Criteria

The inclusion criteria in the current study were: using 0.125% atropine eye drops (Tropine Eye Drops; Aseptic Innovative Medicine Co., Ltd., Taoyuan, Taiwan) in both eyes for more than 1 year; all AL and SEq measurement time points taken within the age range of 5–18 years; presenting with AL and cycloplegic refraction at the 6th month of the atropine treatment period (i.e., baseline data); and continuous AL and SEq measurements at least twice after the 6th month of the atropine treatment period and before 18 years of age. To the best of our knowledge, this is the first study to compare the effects between QON and HS frequencies of 0.125% atropine. Therefore, no prior sample size calculation is performed.

### 2.3. Exclusion Criteria

The exclusion criteria in the current study were: poor medication compliance (<80%) regarding atropine doses; any eyes with SEq out of the range of −0.25 D to −6 D; SEq difference in both eyes of >1 D; any eye(s) with astigmatism of >3 D; any eye(s) with combined ocular disease (e.g., cataracts, congenital retinal diseases, amblyopia, and strabismus); any systemic disease that may affect visual acuity and AL growth [18] (e.g., type 1 diabetes mellitus); and any history of receiving other myopia control treatments other than atropine 0.125% (e.g., orthokeratology, myopia control spectacle lens, or soft contact lens). There are reasons that we excluded SEq exceeded the range of −6 D, and the patients had anisometropia. SEq more negative than −6 D indicated that the patient was more intractable for myopia control treatment, which could lead to bias for our study purpose. On the other hand, anisometropic patients may have different AL elongation speeds between the two eyes. This may also lead to bias for our study purpose. Therefore, these patients were all excluded in the current study.

### 2.4. Grouping

This retrospective cohort study included two subject groups. Group 1 comprised children with myopia treated with atropine 0.125% QON, while group 2 comprised children with myopia treated with atropine 0.125% HS. All of the patients were treated with atropine HS initially; however, if intolerable photophobia and near vision disturbance caused complaints, we switched HS atropine to QON atropine treatment. Therefore, the patients in the atropine QON group all came from the atropine HS group, who were intolerant to the side effects of atropine HS. The side effects of the medication were asked at every follow-up time. The patients could all tolerate the adverse effect of this frequency after the switch, and this modification can benefit patients by decreasing adverse effects to increase compliance.

### 2.5. Myopia Monitoring Policy in the ATCFU Database

In the current study, we recorded the children’s baseline cycloplegic refractive error and AL values at their first visit, and we planned to follow up at 3–4-month intervals. If a patient’s AL elongated rapidly or indicated a high risk of progression to high myopia [8], we started myopia control treatment at the second follow-up visit after 3–4 months. Otherwise, children with a low risk of developing high myopia, whose AL elongated slowly compared with children of the same age, were kept under observation until the annual AL changes accelerated to the treatment range [6,8].

### 2.6. Ophthalmic Examinations

The ATCFU database includes detailed ocular examination data from the first visit and regular follow-ups as described above. Uncorrected distance visual acuity and best-corrected visual acuity measurements, slit-lamp anterior segment examination, and measurements of objective refraction errors before and after cycloplegia by using an auto ref/keratometer (ARK-1a/ARK-1; Nidek Co., Ltd., Gamagori, Japan) were performed at the first visit. In detail, for cycloplegia induction, cycloplegia was obtained 1 h after the first instillation of 1% tropicamide (Mydriacyl; Alcon Vision, LLC, Fort Worth, TX, USA) plus 10% phenylephrine hydrochloride (Phenylephrine Eye Drops; Wu Fu Laboratories Co., Ltd., Yilan, Taiwan). Both drops were instilled together every 10 min for three repetitions. Pupil enlargement with no light reflex was confirmed before measurement of the objective refractive error after cycloplegia using the auto ref/keratometer. The mean value of three consecutive measurements was calculated for the final analysis. Cycloplegic refraction was measured directly at further follow-up examinations because these patients had been using atropine with good compliance [11,19].

AL measurements (IOLMaster 500; Carl Zeiss Meditec AG, Jena, Germany) were performed at the first visit, first month, third month, sixth month (baseline), and every 6 months or as necessary.

### 2.7. Outcome Measurements

The two main outcome measurements used in the current study are AL and cycloplegic refraction. Annual AL changes were calculated as ((the difference between the last and the baseline AL, mm)/(the number of days between the last and the baseline AL measurement)) × 365.25. Cycloplegic refraction was presented in SEq, calculated algebraically by adding 1/2 of the cylinder power to the sphere power. Annual SEq changes were calculated as ((the difference between the last and the baseline SEq, diopter)/(the number of days between the last and the baseline SEq measurement)) × 365.25.

### 2.8. Statistical Analysis

The first 6 months of data on outcome measurements (annual AL and SEq changes) were subtracted as washout periods in those children undergoing both atropine QON and HS treatment courses because pharmacological effects in AL shortening and hyperopia shift could last up to 5 months after initiation of atropine treatment [20,21]. The washout period aimed to prevent overestimating the treatment effect and to determine the efficacy of different frequencies for atropine 0.125% treatment more precisely.

The data were analyzed using the SPSS software (version 23.0; IBM Corp, Armonk, NY, USA). Regarding the two-eyes analysis of one patient, the generalized estimating equations (GEE) model was applied to account for the outcome dependency among two eyes in one case [21]. The linking function was identical, and the distribution was normal in the GEE. Independent working correlations and robust standard errors were adopted to obtain the significance of parameters with the lowest corrected quasi-likelihood under the independence model criterion (QICC). GEE was first applied to compare the difference between the two groups. In the correlation analysis, univariate GEE was used to determine the correlations between the dependent variables (annual AL changes and annual SEq changes) and each parameter individually. Significant variables were put into a further multivariable GEE. A two-tailed *p*-value of <0.05 was used as a cut-off value for the significance of all applicable measures.

## 3. Results

### 3.1. Subject Enrollment, Comparison of Demographic Data, and Clinical Characteristics of the Two Groups

**Figure 1:**
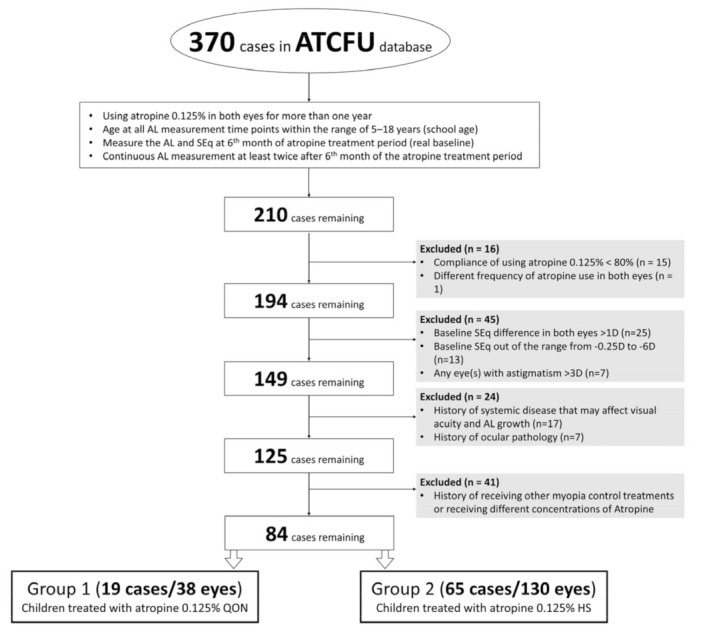
Flow diagram for case selection into the final analysis. ATCFU, atropine continuous follow-up; QON, every other night; HS, once every night; AL, axial length; SEq, spherical equivalent; D, diopters.

The flow diagram for case selection for final analysis is summarized in Figure 1. A total of 38 eyes (19 children) treated with atropine QON and 130 eyes (65 children) treated with atropine HS were included. The baseline characteristics and treatment results are summarized in Table 1. The average baseline ages of groups 1 and 2 were 10.6 years (range, 4.6–13.9 years; median, 11.1 years) and 10.2 years (range, 4.7–16.6 years; median, 9.7 years), respectively. The two groups had no significant difference in baseline characteristics, total follow-up period, or annual change of either AL or SEq (*p* = 0.155 and 0.486, respectively).

### 3.2. Baseline Age Is Negatively Correlated with Annual AL Changes

**Figure 2:**
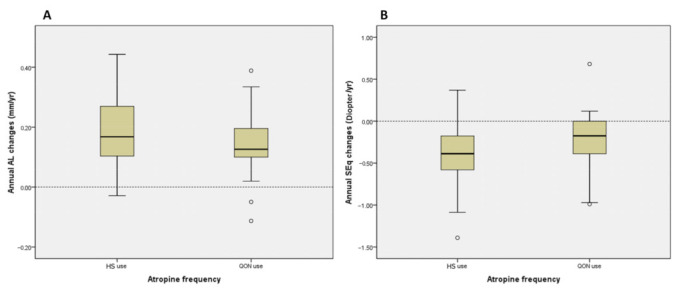
Comparison of annual AL and SEq changes between two groups under different atropine treatment frequencies. (**A**). Distributions of annual AL changes between two groups under different atropine frequencies were not significantly different. (**B**). Distributions of annual SEq changes between two groups under different atropine frequencies were not significantly different. * *p* < 0.001, paired-sample *t*-test. Box plots in panels indicate the minimum, first quartile, median, third quartile, and maximum values. Possible outliers are indicated by “o.” AL, axial length; SEq, spherical equivalent; HS, once every night; QON, every other night.

Figure 2A reveals no remarkable difference in the distributions of annual AL changes during HS and QON atropine treatment. Univariate GEE showed that only younger baseline age (B = −0.020, *p* < 0.001) was significantly correlated with faster annual AL changes (Table 2).

### 3.3. Male Sex Is the Only Factor Correlated with Slower Annual SEq Changes toward Myopia

The distributions of annual SEq changes between the two groups under different treatment protocols of atropine treatment (Figure 2B) demonstrated no remarkable difference. Moreover, the male sex was the only factor significantly associated with slower annual SEq changes toward myopia (B = 0.152, *p* = 0.022) (Table 3).

## 4. Discussion

To the best of our knowledge, this is the first cohort study to directly compare the efficacy of lower-dose atropine with less frequency for myopia control. There were no statistical differences in annual AL and SEq changes between children receiving atropine 0.125% QON and those receiving atropine 0.125% HS, which suggests that the former could be an alternative strategy for children who cannot tolerate the side effects of atropine 0.125% HS [22,23,24].

As lower concentrations of atropine have been proven to be effective and considering that the pharmacological effect of atropine can last for up to 1 week [25], we suggest that atropine 0.125% QON has the potential to be an effective therapy. A previous study also reported that monocular administration of 1% atropine every 3 days in the eye with the longer AL was an effective treatment for myopia and mixed types of anisometropia [26].

To briefly summarize the basic pharmacology of atropine, there are two main adverse effects: mydriasis and cycloplegia [19,27]. The mydriasis effect commences 30 min after exposure, and patients usually recover from the effect 7 to 10 days after cessation of treatment [19,27]. As spending time outdoors is an intervention to prevent or delay myopia development, we encourage children to spend more time outdoors clinically. However, photophobia resulting from mydriasis may decrease the motivation for outdoor activity, especially during the summer [28].

The cycloplegic effect commences 40 min following instillation, with complete recovery in 10 days to 2 weeks after cessation of treatment [19,27]. The effect results in blurred vision at a normal reading distance that may be unfavorable for compliance and convenience because children may require bifocal or multifocal lenses or take their glasses off to aid reading at a closer distance. For the above reasons, it is common to use atropine 0.125% QON in clinical practice to increase the compliance of some patients. Our retrospective study excluded compliance rates of <80% among patients in the beginning (n = 15); all 15 patients are in the HS group. This may hint that the adverse effect of atropine may be lesser in the QOD group, resulting in better compliance.

Aside from these two adverse effects, hyperopic shift with AL decrease has been observed after initiation of atropine treatment for up to 5 months, as shown previously [11,19,20,29]. We excluded data collected within 6 months of treatment to eliminate this effect, which is our washout period. This may not only solve the problem of overestimation of the effect of treatment but also eliminate any bias caused by previous interventions that we may not have been aware of before starting treatment in our practice.

When comparing the study results with the previous ATOM 2 [25] and LAMP 2 studies [12], our outcome measurements regarding annual AL changes and annual SEq changes are compatible with both of these studies, as summarized in Table 4. Notably, compared with the ATOM 2 study, we observed faster AL progression even though our baseline AL is shorter on average and our baseline age is older. This may be due to our washout period preventing an overestimation of the effect of atropine treatment, but it remains an issue that merits further large-scale study for confirmation.

When compared with the effects of atropine at the concentration suggested in the LAMP 2 study (0.05% atropine every day (QD)), our subjects treated with atropine 0.125% HS showed slower AL progression. This finding aligns with the previous conclusion that a higher concentration of atropine has a better treatment effect [12,13,14,15,16,17,18,19,20,21,22,23,24,25,30]. Furthermore, it is evidenced that treatment at even half the frequency could have a similar effect in comparison to atropine, 0.05% QD, even with a washout period to prevent overestimation of the treatment effect (notably, there was no washout period in the LAMP 2 study) [12,25,30]. While this phenomenon might result from the older baseline age or, the shorter baseline AL in our patients, further investigations should be performed to determine the best combination of atropine concentration and frequency for optimum control of myopia.

Younger baseline age is associated with more annual AL changes (Table 2), with a trend of faster myopia progression (Table 3). This finding is consistent with those of previous studies, which showed greater myopia progression rates were expected at younger ages than at older ages [5,8]. The accelerating AL growth may be diminished by atropine treatment that prevents further pathological growth in AL. However, a trend in physiological AL growth is still largely correlated with age [12,18,22,31].

Girls had faster myopia progression in the current study. The role of sex is significant for myopic progression in SEq (β = 0.152, *p* = 0.022) but has no significance in AL (β = −0.032, *p* = 0.211). Differences in SEq have been found in some [32,33,34] but not all studies [35,36] and could be associated with the shorter duration of outdoor activity in girls [10,37]. The situation in the current study may also be explained by small sample sizes or selection bias. On the other hand, myopia progression can also be influenced by environmental and genetic factors yet to be determined. Further extensive research is necessary to clarify the role of sex in the development of myopia.

There are some limitations to the present study, including its retrospective design, small sample size, and lack of detailed genetic and environmental information. It is a fact that patients in the atropine QON group usually came from the populations that were intolerant to the side effect of atropine HS; therefore, there is an imbalanced sample distribution. This could result in potential selection bias. Further prospective studies should be performed to confirm the results. Moreover, we analyzed two eyes from each patient and corrected the dependency of the outcome for the two eyes using the GEE model. It merits future prospective studies to address these limitations and to confirm the study results.

## 5. Conclusions

In conclusion, the efficacy of myopia control by 0.125% atropine is similar between QON and HS frequencies. The results were confirmed by both AL elongation and cycloplegic refraction increase as outcome measures. This observation suggests that the use of atropine 0.125% QON can be an alternative strategy for children who have poorly tolerated the side effects of HS frequency. Further large-scale prospective studies should be performed to confirm the results of the current pilot study.

## Figures and Tables

**Table 1:** Baseline characteristics and treatment results for the studied subjects.

Parameter		Total	QON	HS	*p* Value
Eye number	[n]	168	38	130	
Male sex	(%)	48.8	52.6	47.7	0.592 ^†^
Baseline age (median)	(yr)	10.3 ± 2.6 (10.1)	10.6 ± 2.4 (11.1)	10.2 ± 2.6 (9.7)	0.320 ^a^
Total follow-up period ^‡^	(m)	16.9 ± 9.1	15.6 ± 11.4	17.3 ± 8.3	0.392 ^a^
Baseline AL	(mm)	23.93 ± 1.01	23.81 ± 0.96	23.97 ± 1.02	0.396 ^a^
Baseline SEq	(D)	−1.46 ± 1.46	−1.56 ± 1.65	−1.43 ± 1.41	0.633 ^a^
Annual AL changes ^§^	(mm/yr)	0.18 ± 0.12	0.16 ± 0.10	0.18 ± 0.12	0.155 ^a^
Annual SEq changes ^¶^	(D/yr)	−0.33 ± 0.38	−0.29 ± 0.44	−0.34 ± 0.36	0.486 ^a^

Continuous data are presented as mean ± standard deviation. QON, every other night; HS, once every night; AL, axial length; SEq, spherical equivalent; D, diopters. ^a^ Independent t-test; ^†^ Pearson’s chi-square test; ^‡^ Total follow-up period, months, was calculated as ((the number of days between the last and the baseline AL measurement)/30.44); ^§^ Annual AL changes, mm/year, were calculated as ((the difference between the last and the baseline AL, mm)/(the number of days between the last and the baseline AL measurement)) × 365.25; ^¶^ Annual SEq changes, D/year, were calculated as ((the difference between the last and the baseline SEq, D)/(the number of days between the last and the baseline SEq measurement)) × 365.25.

**Table 2:** Associations between annual AL changes and age, gender, atropine use, and ocular biometrics.

Parameter	Univariate			
		95% CI		
	B	Lower	Upper	Sig.
Male sex	−0.032	−0.083	0.018	0.211
Baseline age (yr)	−0.020	−0.028	−0.013	<0.001 *
Atropine QON use	−0.032	−0.084	0.020	0.233
Total follow-up period (m) ^†^	−0.002	−0.004	0.001	0.061
Baseline AL (mm)	−0.007	−0.035	0.022	0.654
Baseline SEq (D)	−0.003	−0.018	0.013	0.735

Dependent variable: Annual AL changes (mm/year) were calculated as ((the difference between the last and the baseline AL, mm)/(the number of days between the last and the baseline AL measurement)) × 365.25. QON, every other night; AL, axial length; SEq, spherical equivalent; D, diopters; CI, confidence interval. * *p* < 0.05, generalized estimating equation. ^†^ Total follow-up period (in months) was calculated as ((the number of days between the last and the baseline ocular biometrics)/30.44).

**Table 3:** Associations between annual SEq changes and age, gender, atropine use, and ocular biometrics.

Parameter	Univariate			
		95% CI		
	B	Lower	Upper	Sig.
Male sex	0.152	0.022	0.283	0.022 *
Baseline age (yr)	0.022	−0.001	0.045	0.059
Atropine QON use	0.048	−0.128	0.225	0.591
Total follow-up period (m) ^†^	0.001	−0.005	0.007	0.779
Baseline AL (mm)	−0.019	−0.088	0.050	0.594
Baseline SEq (D)	0.011	−0.038	0.060	0.654

Dependent variable: Annual SEq changes (D/year) was calculated as ((the difference between the last and the baseline SEq, D)/(the number of days between the last and the baseline SEq measurement)) × 365.25. QON, every other night; AL, axial length; SEq, spherical equivalent; D, diopters; CI, confidence interval. * *p* < 0.05, generalized estimating equation. ^†^ Total follow-up period (in months) was calculated as ((the number of days between the last and the baseline ocular biometrics)/30.44).

**Table 4:** Comparison of the current study population and main outcome with previous ATOM2 [25] and LAMP2 [12] studies.

Parameter	The Current Study		Chia et al. (ATOM2)	Yam et al. (LAMP2)
	QON	HS	0th–2nd Year	0th–2nd Year
Atropine concentration (%)	0.125	0.125	0.1	0.05
Compliance	>80%	>80%	>80%	>80%
Washout period	6 months	6 months	2 weeks	None
Baseline age (yr)	10.6 ± 2.4	10.2 ± 2.6	9.7 ± 1.6	8.32 ± 1.71
Male sex (%)	52.6	47.7	53.5	53.8
Baseline AL (mm)	23.81 ± 0.96	23.97 ± 1.02	25.1 ± 0.8	24.88 ± 0.91
Annual AL changes (mm/yr)	0.16 ± 0.10	0.18 ± 0.12	0.14 ± 0.14	0.195 ± 0.175
Baseline SEq (D)	−1.56 ± 1.65	−1.43 ± 1.41	−4.5 ± 1.4	−3.93 ± 1.63
Annual SEq changes (D/yr)	−0.29 ± 0.44	−0.34 ± 0.36	−0.19 ± 0.30	−0.275 ± 0.43

QON, every other night; HS, once every night; AL, axial length; SEq, spherical equivalent; D, diopters.

## Data Availability

Data in this study are available upon reasonable request.
